# The association between bedtime at night and diabetes in US adults: Data from National Health and Nutrition Examination Survey (NHANES) 2015-March -2020 pre-pandemic

**DOI:** 10.1371/journal.pone.0287090

**Published:** 2023-06-13

**Authors:** Shayuan Ouyang, Yinghua Su, Ning Ding, Yingjie Su, Liudang He

**Affiliations:** 1 Clinical Nursing Teaching and Research Section, The Second Xiangya Hospital Central South University, Changsha, Hunan, China; 2 Department of Cardiovascular Surgery, The Second Xiangya Hospital Central South University, Changsha, Hunan, China; 3 Department of Emergency Medicine, The Affiliated Changsha Central Hospital, Hengyang Medical School, University of South China, Changsha, Hunan, China; New York University Grossman School of Medicine, UNITED STATES

## Abstract

**Objective:**

The purpose of this study was to investigate the relationship between bedtime at night and the risk of diabetes in adults.

**Methods:**

We extracted data from 14,821 target subjects from the NHANES database for a cross-sectional study. The data on bedtime came from the question in the sleep questionnaire: “What time do you usually fall asleep on weekdays or workdays?”. Diabetes was defined as fasting blood sugar ≥ 126mg/dL, or glycohemoglobin ≥ 6.5%, or 2-hour Oral Glucose Tolerance Test blood sugar ≥ 200mg/dL, or taking hypoglycemic agent and insulin, or self-reported diabetes mellitus. A weighted multivariate logistic regression analysis was conducted to explore the relationship between bedtime at night and diabetes in adults.

**Results:**

From 19:00 to 23:00, a significantly negative association can be found between bedtime and diabetes(OR, 0.91 [95%CI, 0.83, 0.99]). From 23:00 to 02:00, The relationship between the two was positive(OR, 1.07 [95%CI, 0.94, 1.22]), nevertheless, the P values was not statistically significant(p = 0.3524). In subgroup analysis, from 19:00–23:00, the relationship was negative across genders, and in males, the P-values were still statistically significant(p = 0.0414). From 23:00–02:00, the relationship was positive across genders.

**Conclusion:**

Earlier bedtime (before 23:00) increased the risk of developing diabetes. And this effect was not significantly different between male and female. For bedtime between 23:00–2:00, there was a trend of increasing the risk of diabetes as the bedtime was delayed.

## Introduction

Type 2 diabetes is a chronic disease whose incidence is increasing at an alarming rate around the world. In the early 21st century, the International Diabetes Federation (IDF) estimated that there were 151 million adults with type 2 diabetes worldwide [1], and by 2019 this number may reach 463 million [2]. The etiology of diabetes is complex and it is influenced by multiple risk factors, including obesity [3], physical activity [4], smoking [5], alcohol consumption [6], genetic susceptibility [7,8], and sleep factors [9].

Sleep factors have been illuminated to be associated with the development of diabetes in many cross-sectional and prospective studies. A meta-analysis (prospective research) involving 482,502 participants showed a U-shaped relationship between sleep duration and diabetes, with 7–8 hours of sleep per day having the lowest risk of developing diabetes [9]. Another meta-analysis (prospective cohort research) including 5953 subjects showed that obstructive sleep apnea(moderate to severe)was correlated with a higher risk of diabetes compared without obstructive sleep apnea (RR 1.63; 95% confidence interval (CI): 1.09–2.45) [10]. Nevertheless, no research has examined the effect of bedtime on diabetes. Bedtime is a key node in the body’s circadian rhythm. The light signal is transmitted to the suprachiasmatic nucleus through the retina, and then the suprachiasmatic nucleus transmits the signal to the periphery through the autonomic nervous system and endocrine, thereby affecting the behavior and physiological function of the body [11,12]. And related researches show that circadian rhythm disturbance can increase insulin resistance [13]. Therefore, we believe that different bedtimes will have a certain degree of influence on the occurrence of diabetes.

The NHANES database is a national health and nutrition survey of the U.S. population that collects data on a biennial basis. Since 2015, the database has collected data on bedtime in sleep questionnaires. So we intend to extract relevant data from the database to study the effect of bedtime on diabetes.

## Methods

### Study participants

**Fig 1:**
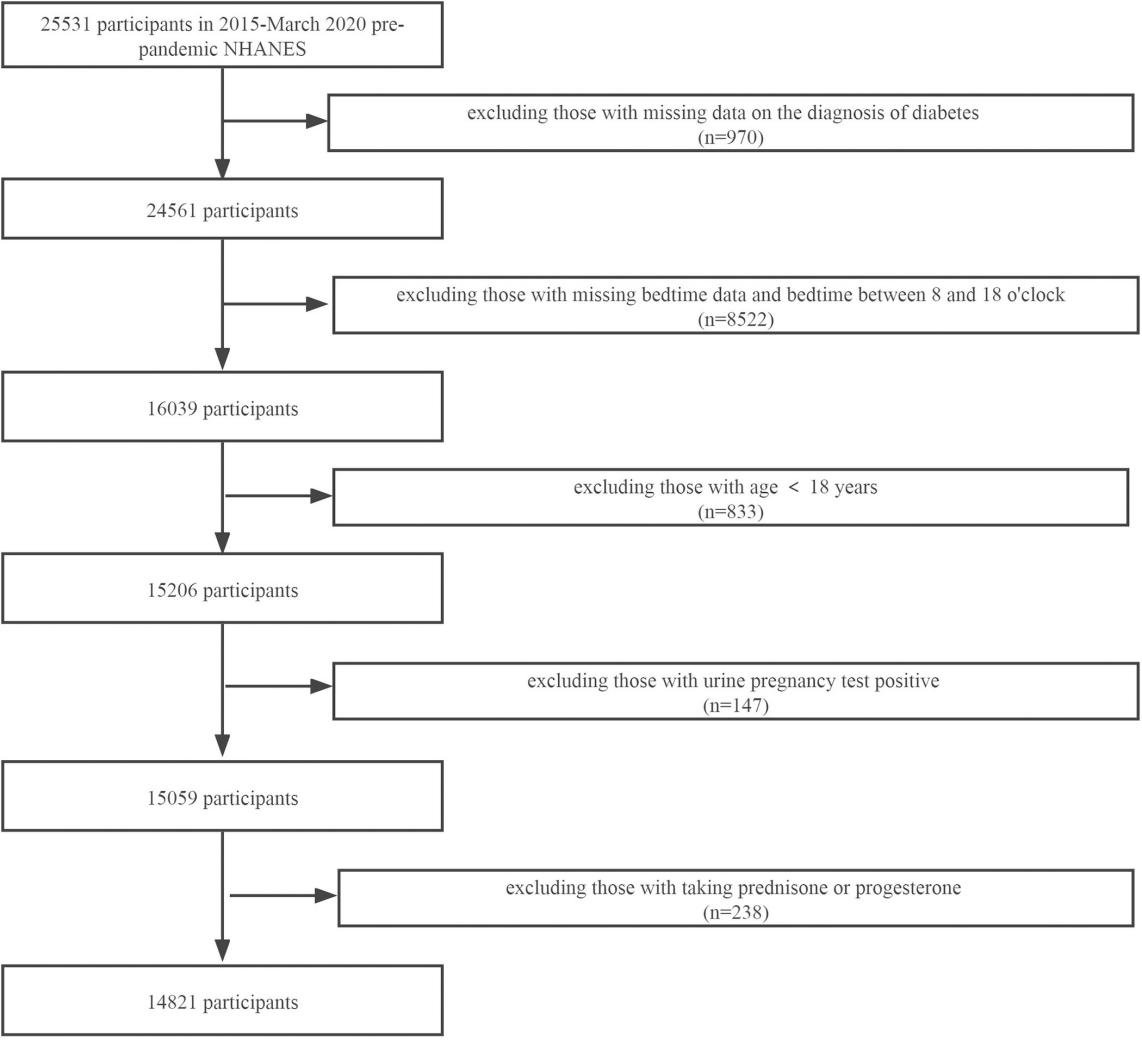
Flowchart of the study design and participants excluded from the study.

Data from 2015-March 2020 pre-pandemic were extracted for analysis. The total sample size is 25,531 subjects, and the final target population is 14,821. Participants will be excluded if they:1) missing data on the diagnosis of diabetes(n = 970); 2) missing bedtime data and bedtime between 8:00 and 18:00(n = 8522); 3) under the age of 18(n = 833); 4) urine pregnancy test positive(n = 147); 5) taking prednisone or progesterone(n = 238). 10710 participants were excluded for the above reasons(**Fig 1**).

### Definition of bedtime and diabetes

Bedtime was the participant’s self-report of the following questions in sleep questionnaire: “What time do you usually fall asleep on weekdays or workdays?”.

Since melatonin was a mediator of endogenous circadian and environmental rhythms in humans **[13].** Its secretion was inhibited during the day and began by the pineal gland when light was diminished **[14]**. Therefore, based on the secretion pattern of melatonin and the time of light exposure, we define the bedtime at night as 19:00–07:00. We use the numbers 0–12 to represent bedtime: 19:00, 20:00, 21:00, 22:00, 23:00, 0:00, 1:00, 2:00, 3:00, 4:00, 5:00, 6:00, 7:00. Then analyzed it as a continuous variable in the data analysis. Diabetes was defined as fasting blood sugar ≥ 7.0mmol/L, or 2-hour Oral Glucose Tolerance Test blood sugar ≥ 11.1mmol/L, or glycohemoglobin ≥ 6.5%, or taking hypoglycemic agent and insulin, or self-reported diabetes mellitus.

### Covariates

Data on potential confounders, such as gender, age, and ethnicity, derived from participant responses to questionnaires, total cholesterol(TC), high density lipoprotein (HDL), uric acid(UA), were derived from laboratory tests performed on fasting blood samples. Alcohol consumption was defined as 12 or more alcohol intakes per year. Smokers are defined as smoking 100 or more cigarettes in their lifetime. We divided smoking status into smoking, non-smoking, and former smoking according to two questions in the smoking questionnaire:“have you smoked at least 100 cigarettes in entire life?”and “do you now smoke cigarettes?”. Total physical activity(TPA) was based on the Global Physical Activity Questionnaire, which included minutes of recreational, occupational, transportation-related physical activity, and was divided into vigorous and moderate categories according to the intensity of each physical activity. Double the time of high-intensity physical activity and add it to the time of moderate-intensity physical activity [15,16]. The sum of the above three types of physical activity was TPA. According to the 2018 Physical Activity Guidelines for Americans [17], a subject was an active participant if his/her TPA minutes were ≥ 150 minutes. Body mass index (BMI) ≥ 30kg/m^2^ was obesity [18], which was calculated by the following formula: weight (in kg)/ height^2^ (in m^2^).

### Definition of other sleep factors

To demonstrate the independent effect of bedtime on diabetes, we also collected other sleep factors into the data analysis, including sleep duration, snort or stop breathing, and trouble sleeping. According to the American Academy of Sleep Medicine Statement on Recommended Sleep Amounts for Adults [19]. Sleep duration was divided into two groups: < 7h, ≥ 7h. The two variables, snort or stop breathing and trouble sleeping factors, were derived from two questions in the sleep questionnaire: “In the past 12 months, how often did you snort, gasp, or stop breathing while you were asleep?”and “Have you told a doctor or other health professional that you had trouble sleeping?”, respectively. and divided into three groups: yes, no, not recorded.

### Statistical analysis

Given the complex survey design (including oversampling), survey non-response, and post-stratification, NHANES created sample weights. A sample weight was assigned to each survey respondent, and when a sample was weighted, its data were representative of the U.S. civilian noninstitutionalized resident population. The weights used for data analysis in this study were mobile examination center(MEC) exam weights and we have adjusted the MEC exam weights of the data for these two periods(15–16, 17-March 2020 pre-pandemic) based on official guideline [20]. We employed weighted multivariate logistic regression to assess the odds ratio (OR) and 95% confidence interval (CI) values of the independent variable to the dependent variable. We used survey-weighted means (95% confidence intervals (CI)) to describe continuous variables, including UA, TC, HDL, and compared clinical characteristics using survey-weighted linear regression. Categorical variables were described using survey-weighted percentage (95% CI), including gender, ethnicity, age, TPA, alcohol consumption, smoke, obesity, sleep duration, snort or stop breathing, trouble sleeping, and compared clinical characteristics using survey-weighted Chi-square test. Missing continuous variables were filled with dummy variables, and missing categorical variables were included in the data analysis as a separate group. We conducted smooth curve fitting to study the nonlinear association between bedtime and diabetes. Our data analysis was performed using EmpowerStats (http://www.empowerstats.com) and statistical package R (http://www.R-project.org). The P value was taken as the cut point of 0.05 to indicate the statistical significance of the results.

## Results

### Characteristics of the study population

**Fig 2:**
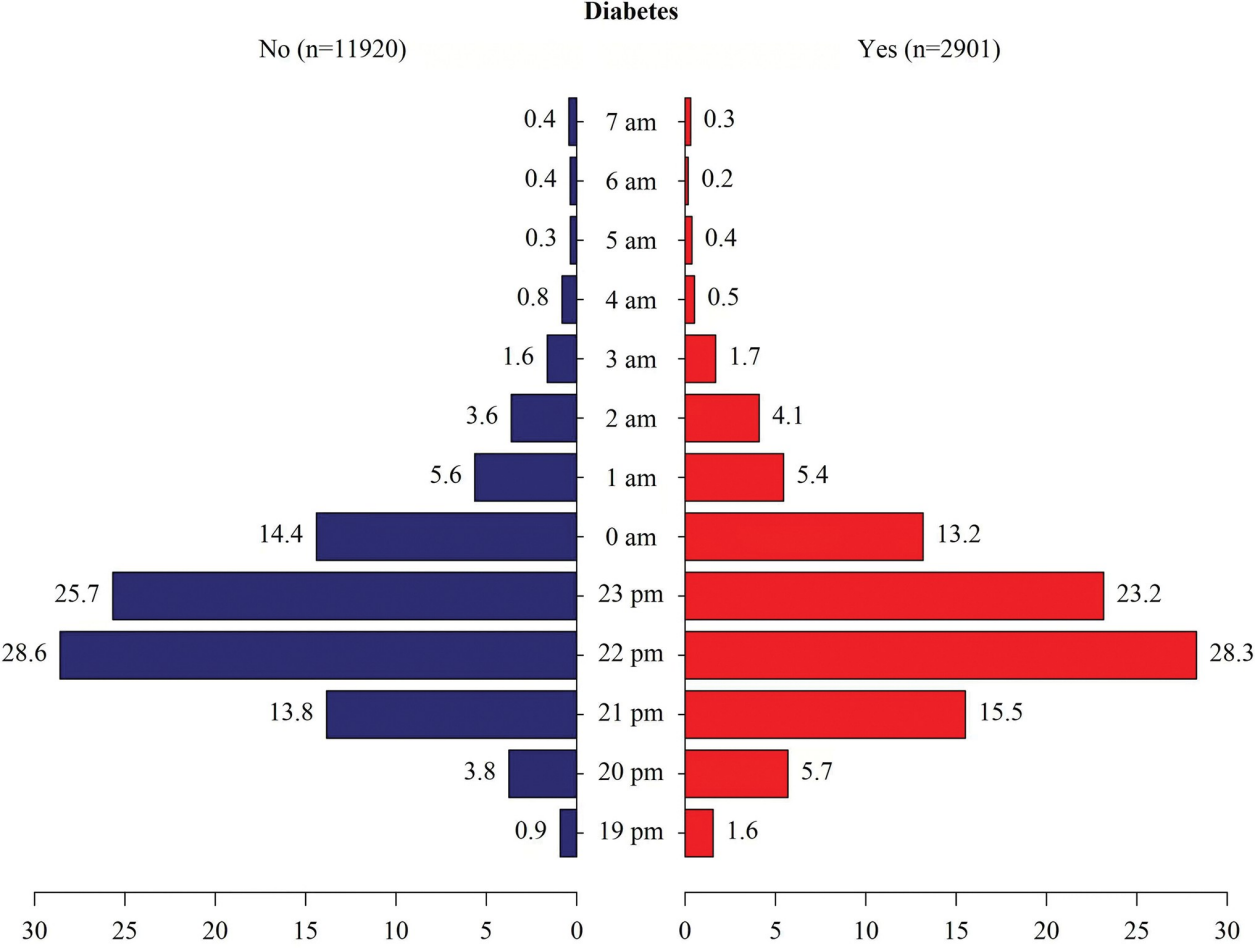
The un-weighted sample proportions of bedtime at different time points based on the presence or absence of diabetes.

Grouping was based on the presence or absence of diabetes, and we described the baseline characteristics of the study subjects in **Table 1**. The sample sizes for diabetes(no) and diabetes(yes) were 11920 and 2901, respectively. In the diabetes(yes) group, the participants were more likely to be male(52.78%), older(54.36%), non-drinkers(47.60%), inactive participants(47.18%), obese(62.33%), and less likely to be smoker(13.93%), and the level of TC, HDL were much more lower. The sleep-related factors of the study subjects were shown in **Table 2**. In the diabetes(yes) group, the proportion of snort or stop breathing and trouble sleeping was 28.67%, 39.83%, respectively. 23.35% subjects with sleep duration less than 7 h. **Fig 2** presented the unweighted distribution of different bedtime points for the study population. We can see from the figure that that most people’s bedtime were distributed at the four time points of 0:00, 23:00, 22:00 and 21:00.

**Table 1:** Description of participants based on the presence or absence of diabetes.

Diabetes	No (n = 11920)	Yes (n = 2901)	P-value
Sociodemographic factors			
Gender			0.0059
Male	5748 (48.21%)	1517 (52.78%)	
Female	6172 (51.79%)	1384 (47.22%)	
Race			0.0001
Mexican American	1622 (8.71%)	495 (10.35%)	
Non-Hispanic White	4164 (63.65%)	856 (58.54%)	
Non-Hispanic Black	2765 (10.79%)	757 (13.06%)	
Other Race	3369 (16.85%)	793 (18.05%)	
Age (years)			<0.0001
18–44	5933 (50.91%)	364 (15.83%)	
45–59	2692 (25.25%)	755 (29.81%)	
≥ 60	3295 (23.84%)	1782 (54.36%)	
Lifestyle factors			
Alcohol consumption			<0.0001
Drinking	5017 (53.27%)	859 (35.34%)	
No drinking	3868 (31.81%)	1336 (47.60%)	
Not recorded	3035 (14.92%)	706 (17.06%)	
Total physical activity			<0.0001
Inactive participants	4212 (30.28%)	1517 (47.18%)	
Active participants	7708 (69.72%)	1384 (52.82%)	
Smoke			<0.0001
Smoking	2179 (17.43%)	422 (13.93%)	
Ex-smoking	2423 (23.30%)	911 (34.56%)	
No smoking	7306 (59.21%)	1565 (51.46%)	
Not recorded	12 (0.06%)	3 (0.05%)	
Metabolic factors			
Obesity			<0.0001
Non-obese	7937 (64.08%)	1311 (37.67%)	
Obese	3983 (35.92%)	1590 (62.33%)	
Total cholesterol(mmol/L)			<0.0001
Mean±SD	4.90 ± 0.95	4.66 ± 1.12	
Median(Q1-Q3)	4.81 (4.24,5.48)	4.58 (3.83,5.30)	
Uric acid (mg/dL)			<0.0001
Mean±SD	5.34 ± 1.31	5.64 ± 1.50	
Median(Q1-Q3)	5.30 (4.40,6.10)	5.42 (4.60,6.60)	
High density lipoprotein(mmol/L)			<0.0001
Mean±SD	1.43 ± 0.40	1.24 ± 0.37	
Median(Q1-Q3)	1.38 (1.14,1.66)	1.16 (0.98,1.40)	

For continuous variables: Survey-weighted mean and survey-weighted median, P-value was by survey-weighted linear regression.

For categorical variables: % was representative of survey-weighted percentages, P-value was by survey-weighted Chi-square test.

**Table 2:** Sleep factors description of 14821 participants included in the present study.

Diabetes	No (n = 11920)	Yes (n = 2901)	P-value
Snort or stop breathing			<0.0001
No	8767 (73.89%)	1821 (63.68%)	
Yes	2571 (22.14%)	826 (28.67%)	
Not recorded	582 (3.97%)	254 (7.65%)	
Trouble sleeping			<0.0001
Yes	2930 (27.86%)	1058 (39.83%)	
No	8981 (72.11%)	1840 (60.11%)	
Not recorded	9 (0.03%)	3 (0.06%)	
Sleep duration(hours)			0.1319
< 7	2717 (21.32%)	718 (23.35%)	
≥ 7	9203 (78.68%)	2183 (76.65%)	

For categorical variables: % was representative of survey-weighted percentages, P-value was by survey-weighted Chi-square test.

### Association between bedtime and diabetes

**Fig 3:**
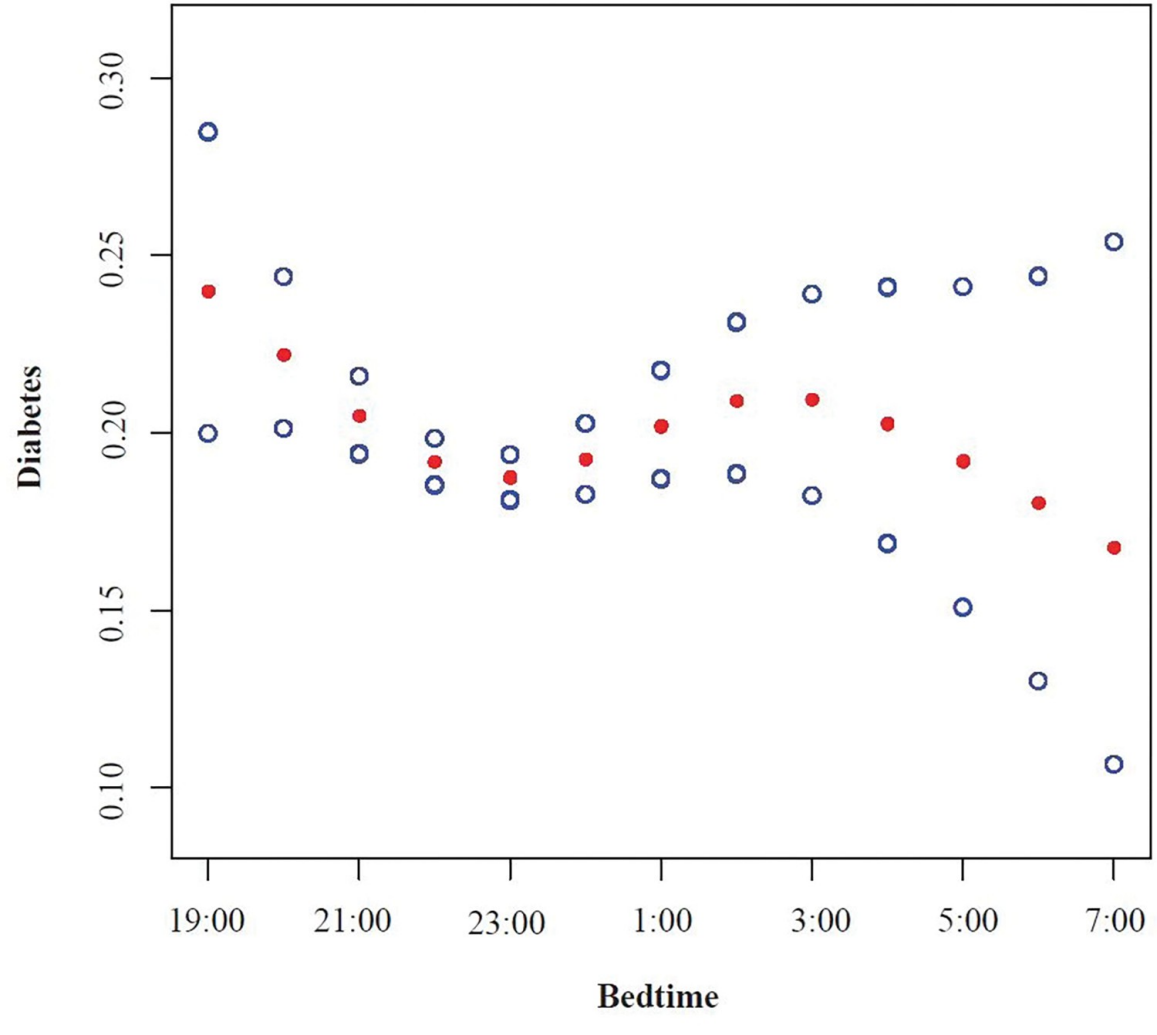
A unweighted smooth curve fitting for the relationship between bedtime and diabetes. Adjust for: Gender, race, age, alcohol consumption, smoke, total physical activity, total cholesterol, uric acid, high density lipoprotein, obesity, sleep duration, trouble sleeping, snort or stop breathing. Red dotted line represents the adjusted effect value, blue dotted line represents the 95% CI.

**Fig 4:**
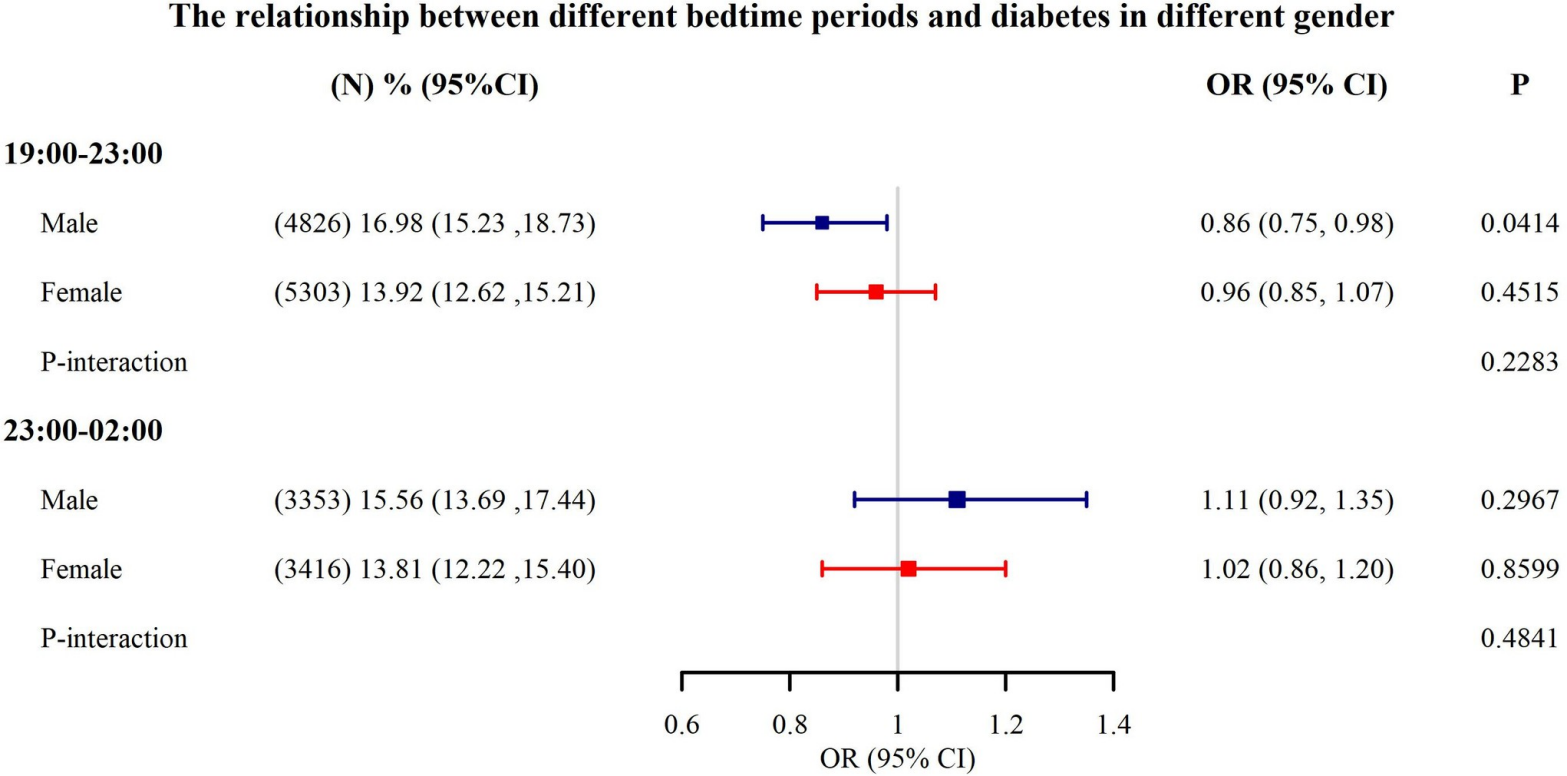
The relationship between different bedtime periods and diabetes in different gender. Adjust for: Race; age, alcohol consumption, smoke, total physical activity, total cholesterol, uric acid, high density lipoprotein, obesity, sleep duration, trouble sleeping, snort or stop breathing. N: Number of observed; % (95%CI): Survey-weighted percentage (95% CI) of diabetes; For diabetes: Survey-weighted OR (95%CI) p-value.

**Table 3** illustrated the results of a weighted multivariate logistic regression analysis between bedtime at night and adult diabetes incidence. In the model I, we adjusted for sleep-related factors: sleep duration, snort or stop breathing, trouble sleeping, into the model, a significantly negative association can be found between bedtime and diabetes(OR, 0.95 [95%CI, 0.92, 0.99]). In Model II, Model III and Model IV, we gradually added social, lifestyle, and metabolic factors into the model adjustment, but the relationship was not statistically significant. The estimates and 95% confident intervals for each variable in Models I-IV can be found in **S1 Table.** To explore the nonlinear relationship between bedtime and diabetes, we performed a smooth curve fitting(**Fig 3**). From the figure, we can see that the relationship between bedtime and diabetes was U-shaped from 19:00 to 02:00, and was lowest at 23:00. However, we can see from Fig 2 that the proportion of sample size at each time point after 3:00 was very small (0.2–1.7%), and it can not reflect the true relationship between bedtime(03:00–07:00) and diabetes. So we took 23:00 as the inflection point, and divided the bedtime into two time periods: 19:00–23:00 and 23:00–02:00, and re-explored the relationship between bedtime and diabetes (**Table 4**). From 19:00 to 23:00, a significantly negative association can be found between bedtime and diabetes(OR, 0.91 [95%CI, 0.83, 0.99]). From 23:00 to 02:00, The relationship between the two was positive(OR, 1.07 [95%CI, 0.94, 1.22]), nevertheless, the P values was not statistically significant(p = 0.3524). We also explored the effect of bedtime on diabetes in different genders(**Fig 4**). From 19:00–23:00, the relationship was negative across genders, and in males, the P-values were still statistically significant(p = 0.0414). From 23:00–02:00, the relationship was positive across genders. And there was no significant interaction of gender on the relationship between bedtime and diabetes at different time periods.

**Table 3:** Relationship between bedtime and diabetes in different models.

Diabetes	(N) % (95%CI)	Model I(OR,95%CI,P)	Model II(OR,95%CI,P)	Model III(OR,95%CI,P)	Model IV(OR,95%CI,P)
Bedtime	(13882) 15.40 (14.45,16.36)	0.95 (0.92, 0.99) 0.0352	1.01 (0.97, 1.05) 0.7075	1.00 (0.96, 1.05) 0.9120	0.99 (0.94, 1.03) 0.5433

N: Number of observed.

% (95%CI): Survey-weighted percentage (95% CI) of diabetes.

For diabetes: Survey-weighted OR (95%CI) p-value.

Model I was adjusted for: Sleep duration, trouble sleeping, snort or stop breathing.

Model II was adjusted for: Gender; race; age in addition to model I.

Model III was adjusted for: Alcohol consumption, smoke, total physical activity in addition model II.

Model IV was adjusted for: Total cholesterol, uric acid, high density lipoprotein, obesity in addition model III.

**Table 4:** The relationship between different bedtime periods and diabetes.

Bedtime	(N) % (95%CI)	OR(95% CI)	P-value
19:00–23:00	(10129) 15.37 (14.31,16.42)	0.91 (0.83, 0.99)	0.0492
23:00–02:00	(6769) 14.69 (13.34,16.05)	1.07 (0.94, 1.22)	0.3524

N: Number of observed.

% (95%CI): Survey-weighted percentage (95% CI) of diabetes.

For diabetes: Survey-weighted OR (95%CI) p-value.

Adjust for gender, age, race, smoking, alcohol consumption, total physical activity, total cholesterol, uric acid, high density lipoprotein, obesity, sleep duration, trouble sleeping, snort or stop breathing.

## Discussion

This research performed on US adult population included 14821 subjects revealed that bedtime was significantly associated with the incidence of diabetes. Earlier bedtime (before 23:00) increased the risk of developing diabetes. And this effect was not significantly different between male and female. For bedtime between 23:00–2:00, although the data was not statistically significant, the risk of developing diabetes tended to increase as bedtime was delayed.

The time of go to bed was a critical node in the sleep-wake cycle and light was the main synchronizer of the central circadian clock(suprachiasmatic nucleus). The suprachiasmatic nucleus then transmitted its signals through the neuroendocrine pathway to peripheral tissues to regulate the circadian clock in peripheral tissues, including: adipose tissue, muscle, pancreas, gut, and liver [11,12,21,22]. These peripheral tissues were involved in glucose metabolism through various pathways. When the biological clock was disturbed, it will lead to the disturbance of glucose metabolism in these tissues, increase insulin resistance, and promote the occurrence and development of diabetes [13]. Melatonin was secreted by the pineal gland and has a distinct circadian rhythm, with higher levels of melatonin during the dark period [23]. and its secretion was inhibited by light [24]. Studies have shown that melatonin inhibited the secretion of insulin from pancreatic β-cells through the melatonin receptor 1b gene, thereby increasing blood sugar levels and increasing the risk of diabetes [25]. This mechanism was a good explanation for our research conclusion: early bedtime increases the risk of diabetes. Earlier bedtime means that the longer the dark period, the longer the high level of melatonin was secreted, and the less insulin was secreted, which may lead to an increased risk of diabetes.

According to our current knowledge, our study should be the first to clarify the relationship between bedtime and diabetes risk. And the conclusions of the study have characteristics that are representative of the population of the United States. We adjusted for diabetes-related risk factors as much as possible, and adjusted for other sleep factors: sleep duration, insomnia, and sleep apnea in order to highlight the effect of bedtime on diabetes. Of course, our study also has certain flaws. Firstly, due to the natural defects of the database, we could not distinguish type 1 diabetes and type 2 diabetes in the diabetic population of this study. In order to make the study conclusions more accurate, we excluded people younger than 18 years, to reduce the number of patients with type 1 diabetes. Similarly, some genetic factors of diabetes we cannot obtain from the database. Secondly, some covariates are based on participants’ self-reports and may deviate from the actual situation.

## Conclusions

Earlier bedtime (before 23:00) increased the risk of developing diabetes. And this effect was not significantly different between male and female. For bedtime between 23:00–2:00, the risk of developing diabetes tended to increase as bedtime was delayed.

## Supporting information

S1 Table The estimate and CI for each variable in the four models. (DOCX)
