# Supplementary material for: The association between bedtime at night and diabetes in US adults: Data from National Health and Nutrition Examination Survey (NHANES) 2015-March -2020 pre-pandemic
Source: PLoS One. 2023 Jun 13;18(6):e0287090. doi: 10.1371/journal.pone.0287090 (PMC10263298; doi:10.1371/journal.pone.0287090)
Supplement: S1 Table — (DOCX) [file pone.0287090.s001.docx]

Supplementary Table 1. The estimate and CI for each variable in the four models.

|  | Model I  (estimate,95%CI) | Model II (estimate,95%CI) | Model III (estimate,95%CI) | Model IV (estimate,95%CI) |
| --- | --- | --- | --- | --- |
| Bedtime | -0.047(0.916,0.995) | 0.008(0.965,1.054) | 0.002(0.961,1.046) | -0.015(0.939,1.033) |
| Sleep duration(hours) |  |  |  |  |
| < 7 | Ref | Ref | Ref | Ref |
| ≥ 7 | -0.126(0.742,1.048) | -0.052(0.795,1.133) | -0.059(0.783,1.135) | -0.039(0.780,1.158) |
| Snort or stop breathing |  |  |  |  |
| No | Ref | Ref | Ref | Ref |
| Yes | 0.332(1.177,1.650) | 0.248(1.060,1.547) | 0.261(1.069,1.577) | 0.123(0.918,1.393) |
| Not recorded | 0.728(1.476,2.908) | 0.458(1.122,2.229) | 0.411(1.064,2.138) | 0.330(0.967,2.001) |
| Trouble sleeping |  |  |  |  |
| Yes | Ref | Ref | Ref | Ref |
| No | -0.487(0.528,0.715) | -0.419(0.564,0.767) | -0.413(0.565,0.775) | -0.323(0.617,0.843) |
| Not recorded | -0.053(0.177,5.087) | -0.620(0.092,3.152) | -0.847(0.060,3.065) | -1.258(0.030,2.731) |
| Gender |  |  |  |  |
| Male | —— | Ref | Ref | Ref |
| Female | —— | -0.294(0.652,0.852) | -0.418(0.571,0.759) | -0.113(0.759,1.051) |
| Age (years) |  |  |  |  |
| 18-44 | —— | Ref | Ref | Ref |
| 45- 59 | —— | 1.357(3.257,4.637) | 1.279(3.015,4.279) | 1.453(3.483,5.251) |
| ≥ 60 | —— | 2.116(6.955,9.910) | 1.912(5.745,7.976) | 2.190(7.499,10.641) |
| Race |  |  |  |  |
| Mexican American | —— | Ref | Ref | Ref |
| Non-Hispanic White | —— | -0.876(0.325,0.534) | -0.775(0.364,0.584) | -0.656(0.412,0.654) |
| Non-Hispanic Black | —— | -0.315(0.584,0.912) | -0.262(0.620,0.954) | -0.096(0.741,1.115) |
| Other Race | —— | -0.396(0.553,0.819) | -0.392(0.556,0.821) | -0.192(0.678,1.004) |
| Smoke |  |  |  |  |
| Smoking | —— | —— | Ref | Ref |
| Ex-smoking | —— | —— | 0.300(1.105,1.651) | 0.250(1.049,1.571) |
| No smoking | —— | —— | 0.058(0.862,1.303) | 0.022(0.819,1.276) |
| Not recorded | —— | —— | -0.680(0.146,1.758) | -0.575(0.168,1.879) |
| Alcohol consumption |  |  |  |  |
| No drinking | —— | —— | Ref | Ref |
| Drinking | —— | —— | 0.602(1.595,2.091) | 0.365(1.250,1.661) |
| Not recorded | —— | —— | 0.575(1.427,2.214) | 0.505(1.333,2.061) |
| Total physical activity |  |  |  |  |
| Inactive participants | —— | —— | Ref | Ref |
| Active participants | —— | —— | -0.397(0.592,0.763) | -0.332(0.639,0.806) |
| TC(mmol/L) | —— | —— | —— | -0.233(0.735,0.854) |
| HDL(mmol/L) | —— | —— | —— | -1.236(0.234,0.361) |
| UA(mg/dL) | —— | —— | —— | -0.029(0.915,1.031) |
| Obesity |  |  |  |  |
| Non-obese | —— | —— | —— | Ref |
| Obese | —— | —— | —— | 0.895(2.135,2.803) |

Abbreviations: TC, Total cholesterol; HDL, high density lipoprotein; UA, Uric acid.
